# Effects of nutrition education using a food-based approach, carbohydrate counting or routine care in type 1 diabetes: 12 months prospective randomized trial

**DOI:** 10.1136/bmjdrc-2020-001971

**Published:** 2021-03-31

**Authors:** Sofia Sterner Isaksson, Margareta Bensow Bacos, Björn Eliasson, Eva Thors Adolfsson, Araz Rawshani, Ulf Lindblad, Johan Jendle, Agneta Berglund, Marcus Lind, Mette Axelsen

**Affiliations:** 1 Department of Molecular and Clinical Medicine, Sahlgrenska Academy, University of Gothenburg, Gothenburg, Sweden; 2 Department of Medicine, NU Hospital Group, Uddevalla, Sweden; 3 Department of Internal Medicine and Clinical Nutrition, Sahlgrenska Academy, University of Gothenburg, Gothenburg, Sweden; 4 Centre for Clinical Research, Region Västmanland, Uppsala University, Uppsala, Sweden; 5 School of Public Health and Community Medicine, University of Gothenburg, Gothenburg, Sweden; 6 Institution of Medical Sciences, Örebro University, Orebro, Sweden

**Keywords:** diabetes mellitus, type 1, diet therapy, education, diet, diabetic

## Abstract

**Introduction:**

Evidence on the effects of structured nutrition education is weak in adults with type 1 diabetes mellitus (T1D) with moderately impaired glycemic control. Objective was to compare the effects of different types of nutrition education programs on glycemic control, cardiovascular risk factors, quality of life, diet quality and food choices in T1D.

**Research design and methods:**

A 12 months randomized controlled study conducted at nine diabetes specialist centers with three parallel arms: (i) a food-based approach (FBA) including foods with low glycemic index or (ii) carbohydrate counting (CC) according to today’s standard practice or (iii) individual sessions according to routine care (RC). The primary end point was difference in glycated hemoglobin A1c (HbA1c) between groups at 12 months.

**Results:**

159 patients were randomized (FBA: 51; CC: 52; RC: 55). Mean (SD) age 48.6 (12.0) years, 57.9% females and mean (SD) HbA1c level 63.9 (7.9) mmol/mol, 8% (0.7%). After 3 months, HbA1c improved in both FBA and CC compared with RC. However, there were no significant differences at 12 months in HbA1c; FBA versus RC (−0.4 mmol/mol (1.3), 0.04% (0.1%)), CC versus RC (−0.8 mmol/mol (1.2), 0.1% (0.1%)), FBA versus CC (0.4 mmol/mol (0.3), 0.04% (0.01%)). At 12 months, intake of legumes, nuts and vegetables was improved in FBA versus CC and RC. FBA also reported higher intake of monounsaturated and polyunsaturated fats compared with RC, and dietary fiber, monounsaturated and polyunsaturated fats compared with CC (all p values <0.05). There were no differences in blood pressure levels, lipids, body weight or quality of life.

**Conclusions:**

Nutrition education using an FBA, CC or RC is equivalent in terms of HbA1c and cardiovascular risk factors in persons with T1D with moderately impaired glycemic control. An FBA had benefits regarding food choices compared with CC and RC.

**Significance of this study**

**What is already known about this subject?**

- Evidence on the effects of structured nutrition education is weak in adults with type 1 diabetes mellitus (T1D) with moderately impaired glycemic control.

**What are the new findings?**

- Nutrition education using a food-based approach, carbohydrate counting or routine care is equivalent in terms of glycated hemoglobin A1c (HbA1c) and cardiovascular risk factors in persons with T1D with moderately impaired glycemic control.
- With a food-based approach the participants increased their intake of legumes, nuts and vegetables compared with carbohydrate counting and routine care.
- A food-based approach also led to higher fiber intake compared with carbohydrate counting, and increased intake of unsaturated fats compared with both other groups.

**How might these results change the focus of research or clinical practice?**

- The results point towards several options in terms of nutrition programs to achieve health goals and quality of life and, thus, more ways to tailor the nutritional management of T1D to each individual’s needs and preferences.

## Introduction

Nutrition is a cornerstone in the prevention of complications in type 1 diabetes mellitus (T1D)^1^ and nutrition education has immediate implications for both blood glucose control and quality of life.^2–6^ Individuals with T1D have an increased risk for cardiovascular disease (CVD) compared with the general population^7 8^ and the risk increases with the number of elevated risk factors including hyperglycemia, hypertension and increased low-density lipoprotein (LDL) levels.^8^ CVD is also the main driver of morbidity and mortality in individuals with T1D.^7^ Intensive diabetes therapy including intensive control of glycemia has beneficial effects on the risk of CVD in T1D.^9–11^ Nutrition education must therefore aim to prevent and reduce cardiovascular risk factors, alongside with optimized blood glucose control and quality of life.

Dietary advice is often delivered in individual counseling sessions. Food-based advice (FBA) is a large component of the practice. The Mediterranean, Dietary Approaches to Stop Hypertension and vegetarian dietary patterns are recommended in the American Diabetes Association’s clinical practice guidelines for people with diabetes.^1^ Dietary advice in TID is mostly based on randomized efficacy studies, often with food supplements such as oils, nuts or low glycemic index (GI) foods, conducted in type 2 diabetes (T2D) populations. Observational studies point towards individual food groups that are protective of cardiovascular disease in individuals with diabetes, including fruit/vegetables/legumes,^12^ fish,^13^ whole grains^14 15^ and nuts/seeds.^16^ The Mediterranean diet, high in olive oil, nuts, vegetables, fruit and cereals has also shown beneficial effects reducing CVD risk in T2D.^17 18^ There is still a gap in evidence from randomized clinical trials in individuals with T1D with respect to different kinds of nutrition education on food choices, nutrient quality and cardiovascular risk factors.

Over the last decades, carbohydrate counting (CC) has received increased endorsement.^1 19^ This has been driven by the superior strength in evidence with respect to benefits of glycated hemoglobin A1c (HbA1c) in individuals with T1D.^1 19 20^ CC is a method for calculating how much insulin to dose together with each meal. The golden standard for CC is a 30-hour group training, modeled from the Dose Adjustment for Normal Eating (DAFNE) trial.^3^ The exact components of the program have not yet been revealed, but similar CC studies have since followed.^4 5 21–23^ However, data unveiling how CC compares with a food-based nutrition education in terms of its effect on glycemic control, diet quality and cardiovascular risk factors in individuals with moderately impaired glycemic control are still lacking.

More options in terms of nutrition programs would be helpful to tailor the nutritional management of T1D to each individual’s needs and preferences.^1^

The aim of the present study therefore was to assess how long-term glycemic control, cardiovascular risk factors, quality of life and diet quality were affected by group education with an FBA, including food with low GI, as compared with group education in CC. A third arm with individual counseling sessions was also included in the trial representing routine care (RC) treatment. The primary end point was difference between groups in HbA1c at 12 months.

### Subjects and methods

This randomized controlled multicenter study was conducted at nine Swedish specialist diabetes centers with two intervention groups and a control group and a follow-up time of 12 months. Enrollment took place between 2013 and 2014. The last subject completed the trial in 2015. Inclusion criteria were T1D diagnosis >3 years, HbA1c 57–78 mmol/mol (7.4%–9.3%), body mass index (BMI) ≤35 kg/m^2^, age 20–70 years. Subjects with serious diabetes complications, pregnancy/planning to become pregnant within the next 12 months, participation in CC education groups lasting >4 hours within the last 2 years and subjects with gluten intolerance or nut allergy were excluded (online supplemental table 1). After having completed the screening process, each center returned the list of screening numbers to the Principal Research Office for a blocked randomization using an electronic generator. Blocked randomization was chosen to enable a balanced number of subjects in the three arms at each center, thereby minimizing risks of center effects. Subjects were allocated to one of three interventions: FBA, CC or RC. The assigned intervention was then placed in individually sealed envelopes, with each subject’s screening number printed on the outside, and sent back to the center to be opened and shown to the subject after baseline measurements were finished. A number of subjects (n=19, 10%) chose not to show up for the baseline measurements and, thus, did not receive their group allocation. These subjects are not included among the cohort of randomized subjects.

10.1136/bmjdrc-2020-001971.supp1 Supplementary data

### Group intervention models

A structured generic intervention model was constructed for both group education programs (CC and FBA). The number of participants was to be ≤8 subjects per group. Both interventions lasted 30 hours in total. There were 10 meetings of 3 hours each, which also contained practical home assignments to be conducted in between meetings. During the first 8 weeks, meetings were held weekly. Two follow-up meetings were then held, at 6 and 9 months. Meetings were held from 18:00 to 21:00 hours. Educational materials were provided to the centers by the Principal Research Office. Dietitians and diabetes specialist nurses from each site participated in a 2-day training workshop prior to the onset of the study.

In both the group programs (FBA and CC) and in the individual program (RC), the dietitians and nurses on each center were instructed to cooperate and exchange expertise with each other, as they normally would.

### Food-based approach: FBA program

In the FBA program groups were led by dietitians. The theme of the program was ‘less guesswork—food choices that work—better health’. One of the home assignments was to progressively incorporate food groups into their diets from a food portfolio. The foods in the portfolio were fish (preferably with high content of fat), mixed nuts and seeds, vegetables, legumes, fruit and berries and whole grains with low GI (online supplemental table 2). The concept was for participants to set their own goals: (a) which foods to incorporate more of, for instance, if fish was not of their liking, they would focus on one of the other groups and (b) which goals to aim for, for example, the number of portions of a specific food to incorporate into their diet during each week. A specific food diary was provided to keep track of results, acknowledge themselves as they made progress and to set new goals. In addition to each person setting their own goals, participants were also informed of ‘goals to strive for’, for example, recommendation of daily intake (online supplemental table 2). The food diary included pictures and weights to inform what ‘one portion’ of each food category represents in practice. The results of each home assignment were discussed at the next group meeting.

10.1136/bmjdrc-2020-001971.supp2 Supplementary data

### Carbohydrate counting: CC program

In the CC program groups were led by diabetes specialist nurses. The theme of the program was ‘less guesswork—more freedom—better health’. The program was inspired by the DAFNE program.^3^ In this modified version, patients were supervised on home assignments to be conducted between meetings, instead of practical exercises during the meetings. The results of each home assignment were discussed at the next group meeting. The advanced CC model from DAFNE was applied, including correction doses. Meal doses of rapid-acting insulin were calculated from the carbohydrate content of the meal according to individual meal ratios.

### Routine care: RC program

In the RC program, also led by diabetes specialist nurses, the instruction to the nurses were regular counseling. Subjects in the RC program were individually scheduled to attend four education sessions by a specialist nurse, with each session lasting up to 1 hour. Sessions took place following each research visit (shortly after baseline measurement, and at 3, 6 and 9 months). Each session had an open agenda, for instance, addressing problems with high or low blood sugar episodes, nutritional issues and/or insulin adjustments. The specialist nurse assisted with education, goal setting and planning to monitor and evaluate outcomes. No structured educational materials were produced. Each center was instructed to use already existing materials.

### Measurements

All measurements were done with standard analytical methods at each center. Height was measured with a stadiometer at baseline. Weight was measured with a calibrated scale, blood pressure were measured after 5–10 min rest and urine samples for measuring microalbuminuria were collected at baseline, 3, 6 and 12 months. HbA1c was measured at baseline, 3, 6, 9 and 12 months. Blood lipids (total cholesterol, high-density lipoprotein (HDL)-cholesterol and LDL-cholesterol, serum triglycerides and high-sensitivity C reactive protein (hs-CRP) were measured at baseline, 3 and 12 months.

A 7-point self-measurement of blood glucose (SMBG) (before and 1.5 hours after main meals and at bedtime) were recorded over 4 days during a given week at baseline, 3, 6 and 12 months. Prandial insulin doses and type of insulin was recorded simultaneously. The numbers of self-reported hypoglycemic events, defined as glucose levels below 3.5 mmol/L, were assessed by interview, at baseline, 3, 6 and 12 months.

Habitual intake of foods included in the food portfolio (vegetables, fruit and berries, legumes, fish, nuts and wholegrain/low GI foods) was recorded in a prospective 4-day food diary at baseline, 3, 6 and 12 months. For appraisal of portion size, subjects used a guide with pictures and weight of standard portions. Nutrient intake was measured by a validated web-based semi-quantitative frequency questionnaire, Meal-Q^24^ at baseline, 3, 6 and 12 months. For logistical reasons, the Food Frequency Questionnaire (FFQ) was only used by the first seven sites because the last two sites started a year later than the others. The following diet variables were analyzed in this study: intake of total energy (kcal), fat, carbohydrates, protein, saturated fatty acid, monounsaturated fatty acid, polyunsaturated fatty acid, n-3, fiber, wholegrain and sucrose.

Quality of life was measured at baseline, 3, 6 and 12 months with Bradley’s Audit of Diabetes Dependent Quality of Life questionnaire.^25^ Question I ‘present quality of life’, question II ‘impact of diabetes on quality of life’ and an average weighted impact of 19 different areas in life and how they are affected by diabetes were analyzed as well as all the separate 19 questions.

### Statistics

The power calculation was based on expected change in HbA1c at 12 months (primary end point). Sixty-four patients were needed in each group at 80% power assuming an SD in HbA1c of 6.3 mmol/mol (0.6%), to detect an HbA1c difference of 4.2 mmol/mol (0.4%), at an alfa level of 5%, two-sided test with an assumed drop-out rate of 25%. A statistical analysis plan was made before the database was locked. The full analysis set (FAS) consisted of all randomized subjects who had at least one follow-up measurement after randomization. Women who became pregnant were excluded from the FAS population. The per-protocol population (PP) consisted of subjects in the FBA and CC groups who attended 6 out of 10 diet education sessions and at least 1 follow-up visit at either 9 or 12 months and those in the RC group attending at least 3 of 4 follow-up visits. Means and SDs, median and quartiles were used for continuous variables with normal and non-normal distributions, respectively and frequencies for categorical/ordinal variables. Differences in continuous variables were evaluated from baseline to each time-point using analysis of covariance with baseline values as the covariate (of the dependent variable) and treatment group as the independent variable. The χ^2^ test was used for categorical variables. Due to skew distribution, Mann-Whitney U test was used for analyses of diet variables and the quality of life questionnaire. Last observation carried forward (LOCF) was used to impute missing data in the FAS population. As a sensitivity analysis, we analyzed all complete cases, that is, without imputation by LOCF. This was done for HbA1c, lipids, blood pressure, body weight, hs-CRP, insulin dose, albumin/creatinine ratio, food diaries and FFQ. It was intended to carry out statistical analyses to explore the effect of interventions on SMBG on four daily SMBG profiles (7 points) recorded at baseline, 3, 6, 9 and 12 months. However, due to large variations in sampling time and high numbers of data missing, data were deemed insufficient in quality to proceed with the planned statistical analyses. SMBG data are thus only narratively presented.

A p value of <0.05 was considered statistically significant. All analyses were performed using SPSS V.21.0 (IBM, released 2012, IBM SPSS Statistics for Windows, Armonk, New York, USA) and R V.2.15.1 (The R Foundation for Statistical Computing).

## Results

### Recruitment

**Figure 1:**
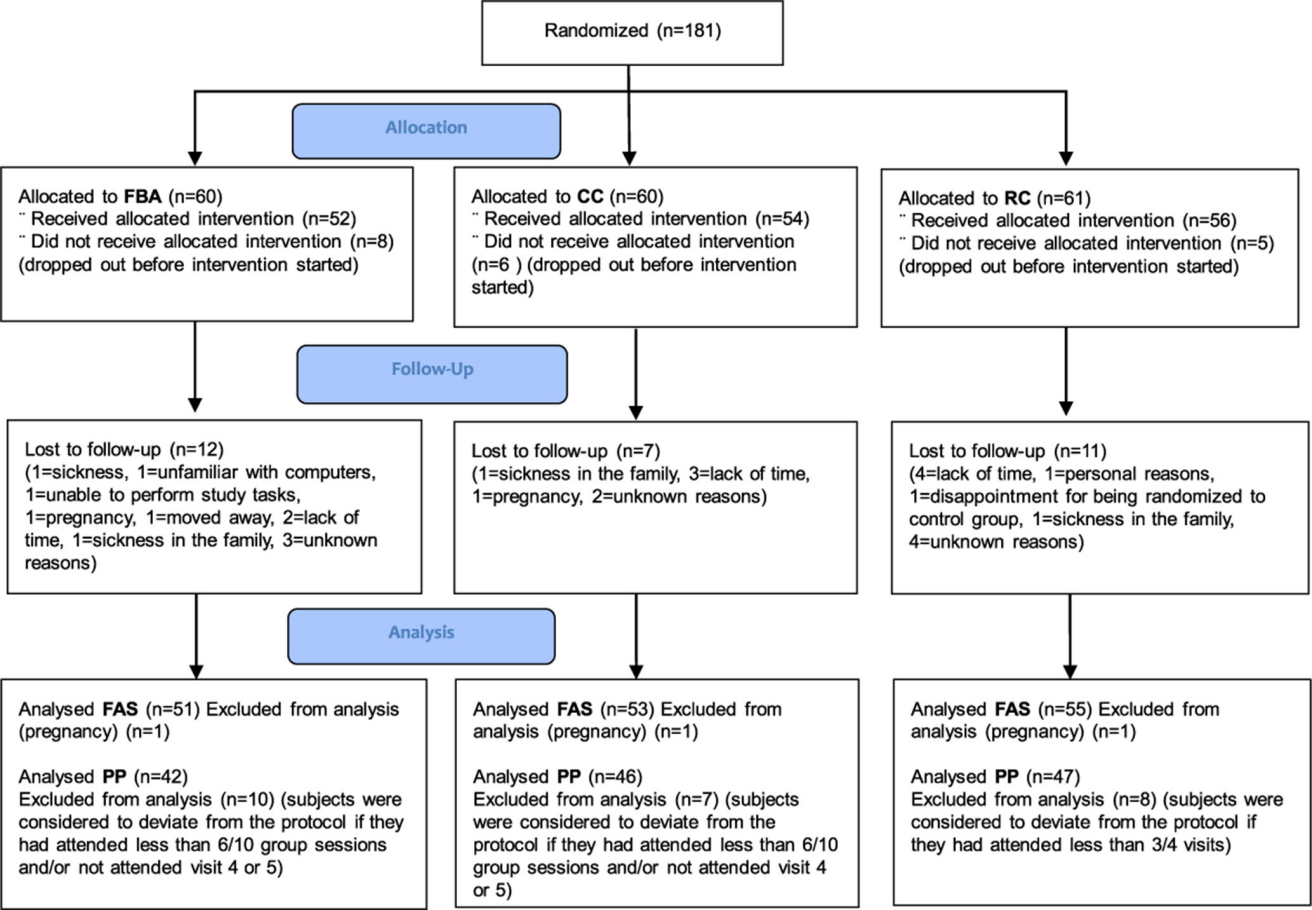
Allocation to education groups, drop-out during 12-month follow-up, and resulting analysis populations. CC, carbohydrate counting; FAS, full analysis set population; FBA, food-based advise; PP, per-protocol population; RC, routine care.

A total of 181 subjects that met the inclusion criteria were randomized. Nineteen subjects did not show up for baseline measurements and the sealed randomization envelopes were not opened. They were therefore not included in the full analysis set (FAS) population. Three subjects became pregnant during the trial and were also excluded from the FAS population. Twenty-seven subjects withdrew during the study period; 23% from FBA group, 14% from CC group and 20% from RC group (p=0.364) (figure 1).

### Baseline characteristics

There were 159 subjects at baseline. Mean age was 48.6 (12.0) years, 57.9% were females. Mean HbA1c was 63.9 (7.9) mmol/mol (8%, 0.7%), mean BMI was 26.4 (3.5) kg/m^2^ and mean diabetes duration was 22.3 (11.6) years. There were no significant differences in baseline characteristics between the three groups (table 1).

**Table 1:** Baseline characteristics of subjects participating in the study

Variables	All subjects n=159	FBA group n=51	CC group n=53	RC group n=55	P value
Age, years	48.6 (12.0)	47.7 (11.5)	49.1 (11.9)	48.9 (12.6)	0.814
Sex					
Females, n (%)	92 (57.9)	27 (52.9)	31 (58.5)	34 (61.8)	0.648
Males, n (%)	67 (42.1)	24 (47.1)	22 (41.5)	21 (38.2)	
Weight, kg	78.9 (14.0)	79.7 (14.5)	77.8 (13.0)	79.3 (14.7)	0.246
BMI, kg/m^2^	26.4 (3.5)	26.2 (3.4)	26.3 (3.5)	26.8 (3.8)	0.606
HbA1c NGSP %	8.0 (0.7)	8.1 (0.7)	7.9 (0.7)	8.0 (0.7)	0.543
HbA1c, mmol/mol	63.9 (7.9)	64.8 (9.0)	63.1 (8.0)	63.7 (6.7)	0.543
Total cholesterol, mmol/L	4.7 (0.9)	4.8 (1.0)	4.7 (0.8)	4.7 (0.9)	0.752
Total cholesterol, mg/dL	182.8 (34.8)	185.6 (38.7)	182.8 (30.9)	182.8 (34.8)	0.752
HDL-cholesterol, mmol/L	1.7 (0.5)	1.7 (0.5)	1.7 (0.5)	1.6 (0.4)	0.226
HDL-cholesterol, mg/dL	65.7 (19.4)	65.7 (19.4)	65.7 (19.4)	61.9 (15.5)	0.226
LDL-cholesterol, mmol/L	2.7 (0.7)	2.9 (0.7)	2.6 (0.6)	2.7 (0.8)	0.121
LDL-cholesterol, mg/dL	104.4 (27.1)	112.1 (27.1)	100.5 (23.2)	104.4 (30.9)	0.121
Triglycerides, mmol/L	1.1 (0.7)	1.1 (0.6)	1.0 (0.5)	1.1 (0.9)	0.815
Triglycerides, mg/dL	97.4 (62.0)	97.4 (53.1)	88.6 (44.3)	97.4 (79.7)	0.815
Systolic blood pressure, mm Hg	127.4 (13.4)	128.6 (13.1)	128.9 (13.7)	124.7 (13.0)	0.192
Diastolic blood pressure, mm Hg	74.6 (9.3)	74.0 (8.1)	75.3 (10.1)	74.3 (9.6)	0.773
Albumin/creatinine ratio, mg/mmol	1.8 (5.4)	2.4 (8.0)	1.2 (2.7)	1.7 (4.1)	0.508
Diabetes duration, years	22.3 (11.6)	24.1 (11.2)	20.8 (11.6)	22.0 (12.0)	0.345
Insulin regimen:					
Injection, n (%)	94 (59.1)	30 (58,8)	33 (62.3)	31 (56.4)	0.822
Pump, n (%)	65 (40.9)	21 (41.2)	20 (37.7)	24 (43.6)	
Insulin dose, IU/kg body weight	0.6 (0.2)	0.5 (0.2)	0.6 (0.2)	0.6 (0.2)	0.115
Lipid lowering medication, n (%)	78 (49.1)	18 (35.3)	30 (56.6)	30 (54.5)	0.057
Antihypertensive medication, n (%)	9 (5.7)	4 (7.8)	4 (7.5)	1 (1.8)	0.312
Smoking, n (%)	13 (8.2)	5 (9.8)	6 (11.3)	2 (3.6)	0.303
Snuff, n (%)	19 (11.9)	4 (7.8)	11 (20.8)	4 (7.3)	0.053
Other nicotine products, n (%)	3 (1.9)	1 (2.3)	2 (4.1)	0 (0.0)	0.380

Data shown as means (SD) unless otherwise stated.

BMI, body mass index; CC, carbohydrate counting; FBA, food-based advice; HbA1c, glycated hemoglobin A1c; HDL, high-density lipoprotein; LDL, low-density lipoprotein; NGSP, National Glycohemoglobin Standardization Program; RC, routine care.

### Primary outcomes

Results of primary and key secondary outcomes in the FAS population are shown in table 2.

**Table 2:** Clinical variables, differences between the groups after 12 months in the full analysis set

	FBA versus RC	CC versus RC	FBA versus CC
HbA1c NGSP %	0.0 (0.1) p=0.792 7.8 (0.7) vs 7.9 (0.8)* n=40 vs 45	−0.1 (0.1) p=0.522 7.8 (0.7) vs 7.9 (0.8)* n=48 vs 45	0.0 (0.0) p=0.754 7.8 (0.7) vs 7.8 (0.7)* n=40 vs 48
HbA1c, mmol/mol	−0.4 (1.3) 61.8 (7.9) vs 62.7 (8.7)* p=0.792 n=40 vs 45	−0.8 (1.2) 61.8 (7.3) vs 62.7 (8.7)* p=0.522 n=48 vs 45	0.4 (0.3) 61.8 (7.9) vs 61.8 (7.3)* p=0.754 n=40 vs 48
Total cholesterol, mmol/L	0.09 (0.06) p=0.131 n=40 vs 46	0.05 (0.06) p=0.376 n=48 vs 46	0.01 (0.06) p=0.846 n=40 vs 48
HDL cholesterol, mmol/L	−0.01 (0.02) p=0.658 n=40 vs 46	0.00 (0.02) p=0.962 n=48 vs 46	−0.01 (0.02) p=0.547 n=40 vs 48
LDL cholesterol, mmol/L	0.04 (0.04) p=0284 n=40 vs 46	0.07 (0.05) p=0.159 n=48 vs 46	−0.04 (0.05) p=0.400 n=40 vs 48
Non-HDL cholesterol, mmol/L	0.08 (0.05) p=0.107 n=40 vs 46	0.05 (0.05) p=0.328 n=48 vs 46	0.02 (0.05) p=0.610 n=40 vs 48
Triglycerides, mmol/L	−0.03 (0.04) p=0.418 n=40 vs 44	−0.08 (0.04) p=0.054 n=48 vs 44	0.05 (0.04) p=0.194 n=40 vs 48
Systolic blood pressure, mm Hg	−0.42 (1.36) p=0.756 n=40 vs 45	−0.14 (1.24) p=0.913 n=48 vs 45	0.12 (1.34) p=0.928 n=40 vs 48
Diastolic blood pressure. mm Hg	0.12 (0.86) p=0.887 n=40 vs 45	−0.14 (0.84) p=0.867 n=48 vs 45	0.12 (0.88) p=0.888 n=40 vs 48
Body weight, kg	−0.23 (0.5) p=0.680 n=40 vs 45	−0.22 (0.60) p=0.713 n=48 vs 45	0.05 (0.56) p=0.935 n=40 vs 48
hs-CRP, mg/L	0.19 (0.35) p=0.586 n=41 vs 46	0.35 (0.32) p=0.278 n=47 vs 46	−0.13 (0.39) p=0.735 n=41 vs 47
Insulin dose, IU/kg body weight	0.03 (0.02) p=0.079 n=37 vs 42	0.01 (0.02) p=0.625 n=44 vs 42	0.03 (0.02) p=0.161 n=37 vs 44
Hypoglycemia Number of events per month	0.39 (0.06) p=0.000 n=35 vs 42	0.05 (0.07) p=0.437 n=44 vs 42	0.35 (0.06) p=<0.000 n=35 vs 44
Albumin/Creatinine ratio, mg/mmol	−0.73 (0.76) p=0.339 n=40 vs 41	−0.00 (0.78) p=0.995 n=48 vs 41	−0.32 (0.82) p=0.699 n=40 vs 48

Data shown as means (SD) unless otherwise stated. All analyses were adjusted for baseline values. The analysis of lipids and blood pressure does not include data from subjects who changed the dose or type of cholesterol/hypertension-lowering medication during the study period.

*Mean (SD) HbA1c at 12 months.

CC, carbohydrate counting; FBA, food-based advice; HbA1c, glycated hemoglobin A1c; HDL, high-density lipoprotein; hs-CRP, high-sensitivity C reactive protein; LDL, low-density lipoprotein; NGSP, National Glycohemoglobin Standardization Program; RC, routine care.

**Figure 2:**
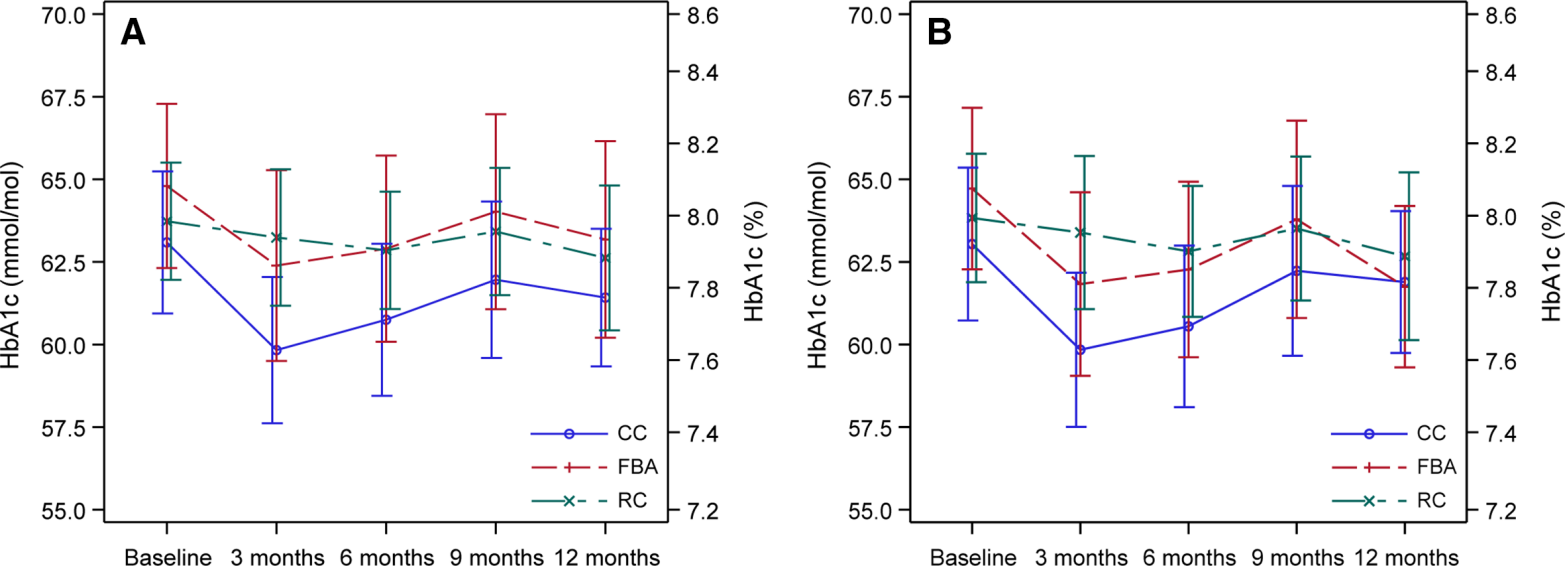
Glycated hemoglobin A1c (HbA1c) before and after different types of nutrition education in type 1 diabetes. (A) Full analysis set population, (B) per-protocol population. Data presented as means and 95% CI. CC, carbohydrate counting; FBA, food-based advise; RC, routine care.

Differences between the three groups in change from baseline to months 3, 6, 9 and 12 were analyzed in both FAS population and PP population. There were no statistically significant differences in HbA1c between the groups after 12 months nor in the FAS or the PP population, nor at 6 or 9 months (figure 2). After 3 months, HbA1c significantly decreased in the CC group compared with RC group both in the FAS analysis (−2.9 mmol/mol (1.0), 0.3% (0.1%), p=0.0057) and the PP analysis (−2.8 mmol/mol (1.1), 0.3% (0.1%), p=0.0144), and also in the FBA group compared with the RC group in the PP population (−3.0 mmol/mol (1.2), 0.3% (0.1%), p=0.0171), although it was less and non-significant in the FAS population (−1.8 mmol/mol (1.1), 0.2%, (0.1%) p=0.111).

### Key secondary outcomes

No significant differences were seen between the groups at 12 months in body weight, blood pressure, albuminuria, total cholesterol, HDL-cholesterol, LDL-cholesterol, non-HDL-cholesterol levels or hs-CRP (table 2), nor at 3 or 6 months. A decreased mean triglyceride level of −0.18 (0.08) mmol/L, −15.9 mg/L (7.09), p=0.041 was seen in the CC compared with the RC group at 6 months in FAS population.

### Glucose profiles

The SMBG profiles from the FAS population indicated lower glucose levels for CC group at all time points, and especially after 3 months. There was also an indication of reduction for FBA group fasting, and after meals (online supplemental figure 1).

10.1136/bmjdrc-2020-001971.supp3 Supplementary data

### Hypoglycemia

At 12 months, the FBA group had an increased mean number of mild self-reported hypoglycemic events compared with both CC and RC groups in the FAS population by 0.4 (0.06) events per month (p<0.001) (table 2).

### Quality of life

There were no statistically significant differences between groups in ‘present quality of life’ or in the ‘overall quality of life’ score at 3, 6 or 12 months in neither FAS nor PP population. Differences were seen in quality of life at 3 months for single questions in the FAS population. The question “If I did not have diabetes, my quality of life would be” had improved in FBA compared with CC (p=0.045). Question 1 ‘leisure activities’ had improved in RC compared with CC (p=0.029) and question 3 ‘travel’ CC had improved compared with RC (p=0.044). In question 8, ‘personal relationships’ both RC and FBA had improved their score compared with CC (p=0.022, p=0.023, respectively). In question 18, ‘freedom to eat’ the score had improved more in RC compared with CC (p=0.006), and there was also a trend in the PP population that FBA had improved this score compared with CC (p=0.05). There were no statistically significant differences between groups in ‘present quality of life’ or in the ‘overall quality of life score’, or in separate questions regarding different life areas was found at 12 months, in either the FAS or PP population.

### Food Frequency Questionnaire (Meal-Q)

There were 124 subjects with nutrient data at baseline from the FFQ. Median energy intake for all subjects was 1700 kcal/day, intake of carbohydrates in per cent of energy was 43 E%, protein 17 E%, total fat 33 E%, saturated fat 12 E% and mean fiber intake 21 g/day Baseline data and differences in dietary intake between groups at 12 months in the FAS population are shown in table 3.

**Table 3:** Baseline data and differences in dietary intake between groups at baseline and after 12 months in the full analysis set

	Baseline			Month 12		
	FBA	CC	RC	FBA	CC	RC
Energy (kcal)	1636 (1321–2136) n=42	1513* (1328–1906) n=41	1821* (1472–2447) n=41	0 (–156–216) n=42	−148 (–329–50) n=41	−94 (–555–85) n=41
Carbohydrates (g)	167† (131–222) n=42	177* (126–207) n=41	202†* (159–202) n=41	0 (–26–17) n=42	−14 (–54–7) n=41	−15 (–64–15) n=41
Protein (g)	71 (61–88) n=42	67* (56–82) n=41	80* (66–106) n=41	0 (–6–12) n=42	−4 (–13–4) n=41	−4 (–23–6) n=41
Fat (g)	65 (49–87) n=42	55* (48–73) n=41	64* (55–95) n=41	0‡ (–2–14) n=42	−5‡ (–12–4) n=41	−3 (–18–7) n=41
SFA (g)	25 (17–33) n=42	21 (19–29) n=41	24 (19–34) n=41	0 (–6–3) n=42	–2 (–4–3) n=41	−2 (–8–1) n=41
MUFA (g)	24‡ (18–32) n=42	20‡* (18–27) n=41	24* (21–33) n=41	0‡† (–1–5) n=42	−2‡ (–5–2) n=41	−1† (–8–2) n=41
PUFA (g)	11 (7–15) n=42	10 (8–15) n=41	12 (9–18) n=41	2‡† (0–5) n=42	0‡ (–3–1) n=41	−1† (–4–2) n=41
n-3 (g)	0.2 (0.2–0.5) n=42	0.2 (0.1–0.5) n=41	0.2 (0.1–0.5) n=41	0.0 (–0.1–0.1) n=42	0.0 (–0.2–0.0) n=41	0.0 (–0.1–0.1) n=41
Sucrose (g)	26 (17–36) n=42	27 (19–40) n=41	30 (19–45) n=41	0† (–3–10) n=42	0 (–7–3) n=41	−2† (–16–2) n=41
Fiber (g)	21† (15–25) n=42	19 (14–27) n=41	23† (18–23) n=41	0‡ (–3–7) n=42	−2‡ (–6–0) n=41	0 (–7–2) n=41
Wholegrain (g)	49 (26–73) n=42	54 (28–74) n=40	65 (39–99) n=41	0 (–16–14) n=42	−5 (–27–8) n=40	8 (–23–20) n=41
Legumes (portions/day)	0.0 (0.0–0.2) n=42	0.0 (0.0–0.3) n=47	0.0 (0.0–0.3) n=50	0.3 (0.0–0.5)‡† n=42	0.0 (0.0–0.3)‡ n=47	0.0 (0.0–0.3)† n=50
Nuts, seeds and almond (portions/day)	0.3 (0.0–0.6) n=42	0.5* (0.0–0.9) n=47	0.1* (0.0–0.5) n=50	0.2‡† (0.0–0.8) n=42	0.0 (−0.4–0.0)‡ n=46	0.0 (0.0–0.3)† n=50
Vegetables and root vegetables (portions/day)	2.1 (1.4–2.3) n=42	1.8 (1.0–2.3) n=47	2.0 (1.0–2.9) n=49	1.8 (0.0–9.2)‡† n=42	0.0 (–4.4–5.3)‡ n=47	0.0 (–5.3–0.9)† n=49
Fruit and berries (portions/day)	1.1 (0.9–1.5) n=42	1.3 (0.8–1.8) n=46	1.0 (0.5–2.0) n=50	0.1 (0.0–0.7) n=42	0.0 (–0.4–0.4) n=46	0.0 (0.0–0.5) n=50
Fish (portions/day)	0.4 (0.2–0.5) n=42	0.3 (0.0–0.5) n=47	0.3 (0.2–0.6) n=50	0.0 (–0.1–0.3) n=42	0.0 (–0.3–0.3) n=47	0.0 (–0.2–0.3) n=50
Wholegrain products (portions/day)	2.0 (1.3–3.0) n=42	1.5 (1.0–2.8) n=47	2.0 (1.0–3.1) n=50	−1.8 (–7.0–0.0) n=42	0.0 (–5.3–0.0) n=47	0.0 (–5.7–0.0) n=50

Baseline data and differences in dietary intake between groups in FAS analysis expressed in medians and quartiles 1 and 3 at baseline and after 12 months.

*CC versus RC p≤0.05.

†FBA versus RC p<0.05.

‡FBA versus CC p<0.05.

CC, carbohydrate counting; FAS, full analysis set; FBA, food-based advice; MUFA, monounsaturated fatty acid; PUFA, polyunsaturated fatty acid; RC, routine care; SFA, saturated fatty acid.

There were no baseline differences between groups in energy, fat, protein, sucrose or wholegrain intake per gram, but in carbohydrate (p=0.014) and fiber (p=0.044) intake (table 3). FBA group significantly increased their fiber intake at 12 months compared with CC group in both FAS and PP population. They also increased their fiber, and monounsaturated and polyunsaturated fat intake compared with RC group (online supplemental figure 2) in both FAS and PP population. Regarding energy intake (kcal), sucrose, protein, total fat, omega-3 fat, wholegrain and total carbohydrates, there were some differences in intake at different time points between groups, but they were not consistent during the 12 months and not consistent in either FAS or PP population (table 3, online supplemental table 3 and table 4).

10.1136/bmjdrc-2020-001971.supp4 Supplementary data

10.1136/bmjdrc-2020-001971.supp5 Supplementary data

10.1136/bmjdrc-2020-001971.supp6 Supplementary data

### Food diary

FBA significantly increased their intake (portions) of legumes, nuts and seeds at all time points compared with CC and RC in both FAS and PP population. They also had increased their intake (portions) of vegetables and root vegetables compared with RC and CC at 12 months in the FAS population (table 3). Regarding portions of wholegrain products with low GI, all groups decreased their intake at all time points in the FAS population.

### Sensitivity analyses

With regard to the sensitivity analysis only using the actual values at each time point, they showed similar findings in comparison with those obtained using data imputed by means of LOCF. The difference between groups in HbA1c at 12 months, which was the primary end point, was: FBA compared with RC; 0.5 (1.9) mmol/mol, 0.1 (0.2) % p=0.765, CC compared with RC; −1.2 (1.5) mmol/mol, 0.1 (0.1) % p=0.438, FBA compared with CC; 1.8 (1.8) mmol/mol, 0.2 (0.2) % p=0.341. The effect was also similar at all the other time points (online supplemental table 5). This was also the case for the other sensitivity analyses on lipids, blood pressure, body weight, hs-CRP, insulin dose, albumin/creatinine ratio, food diaries and FFQ (data not shown).

10.1136/bmjdrc-2020-001971.supp7 Supplementary data

## Discussion

In this study, we introduced a novel type of nutrition education that was a group program with FBA, and that was similar in terms of duration and home assignments compared with CC group. When comparing these two different group educations, FBA and CC, with RC, we could see short-term positive effects on HbA1c in both group educations but after 12 months no effects remained. This was supported by the SMBG curves that indicated lower plasma glucose levels premeal and postmeal for especially CC group at 3 months, and for FBA group at some time points.

The method behind the CC education was inspired by the DAFNE trial.^3^ The DAFNE trial compared group education in CC with a control group on a waiting list. They were able to show improved HbA1c and quality of life that persisted over a year. This could not be seen in our study. One explanation could be the difference in glycemic control at baseline. In DAFNE, the subjects had very poor glycemic control, while in this study they had a moderate glycemic control. This is supported by another study with the same approach as DAFNE, but with subjects with moderate glycemic control that also failed to see any effects on HbA1c.^23^ Another aspect is that FBA and RC received more active comparison treatment compared with the control group in DAFNE. In DAFNE, they also had positive results in ‘present quality of life’ and ‘freedom to eat’. In this study, we did not see that effect in the CC group; it was instead the RC group that had better scores than CC after 3 months and a trend towards better scores in FBA as well. However, the effect did not persist. It is possible that the interventions were considered too hard to follow for the participants so that the intervention affected their quality of life in a negative way. A meta-analysis comparing CC with usual care or alternate dietary advice^26^ did not show any significant improvement in HbA1c either. Although a problem when comparing results from earlier studies and meta-analyses comparing CC with control groups or other dietary interventions is the heterogeneity in both design of the groups (eg, different kinds of CC techniques) and participants (eg, glycemic control at baseline).^22 26^

The FBA group improved their diet quality and food choices (more fiber, unsaturated fats, nuts, legumes and vegetables) compared with both CC and RC, an effect that persisted after 12 months. These reported changes were well in line with the advice given during group education. One unexpected finding was that all three groups decreased their intake (in portions, measured by food diary) of wholegrain products with low GI, this even though the FBA group was encouraged to increase their intake. The Meal-Q analyses showed contradictory results in that FBA group increased wholegrains and fiber intake as measured by FFQ. One explanation could possibly be that in the FBA group they had exchanged processed carbohydrates for wholegrains instead of increasing portion sizes.

Although subjects in FBA group converted to more healthy foods, this change evidently was not of sufficient magnitude to influence cardiovascular risk factors including HbA1c, lipids and blood pressure. In the Prevención con Dieta Mediterránea trial,^17^ a Mediterranean style diet with addition of olive oil or nuts showed positive effects on morbidity and mortality in CVD in T2D. In that study, the participants increased their intake of fish and legumes with 0.3 and 0.4 portions per week, respectively, and with 0.9 or 6 portions of nuts per week (depending on intervention group), to be compared with an increase in portions in the current study of 0.3 portions of nuts and legumes, respectively per day (2.1 portions per week) and a fish intake that only increased significant at 6 months (0.2 portions per day=1.4 portions per week in the FAS population). This indicates a possibility that these kinds of changes in the diet could have a more long-term effect on CVD that could not be detected in this study.

There are several implications from the current study. Diets including more fiber and unsaturated fats are viewed as being essential for cardiovascular prevention.^1 15 27^ Since most individuals with T1D have an increased cardiovascular risk, it is important to reduce this as much as possible. Well-known factors are regulation of glycemic control, lipids and blood pressure levels, and support through a diet program such as FBA is likely essential for certain patient groups. Another implication is that CC, although possibly suitable for some patient groups, does not solve the major problem of hyperglycemia in all subjects with T1D. It is possible that a combination of education in CC, together with the new technique (continuous glucose monitoring (CGM), insulin pumps) and education in healthy eating with FBA could be a more complete and effective method.

Strengths of the current study include the multicenter randomized design and follow-up period of 12 months testing the different group educations in everyday settings. Moreover, several risk factors were examined together with quality of life, diet quality and food choices.

To our knowledge, there is no other study on adults with T1D comparing CC with a structured nutrition education, which is food based, given in equal amount of treatment hours, in addition to a control group. Limitations include documentation of hypoglycemia, which was based on self-estimates and not actual glucose levels. Although we excluded subjects having received education regarding CC the previous years before study inclusion, it is possible that certain subjects could have taken part in other dietary programs before the start of the study. It is also noteworthy that diabetes technology has rapidly emerged and considerably more subjects with T1D today use CGM compared with during the study period. It is a limitation that CGM was not used in this study in order to characterize, in greater detail, the effects on glycemia including time in hypoglycemia, glucose variability and periods with very high glucose levels. Furthermore, as is the case today when most individuals with T1D use CGM in Sweden, where the study was carried out, it can possibly motivate to greater life-style changes by directly visualizing the effects via continuously reported glucose values. The study was not blinded because this was not possible with this kind of interventions.

The data show that CC, an FBA with low GI and RC give equivalent results in terms of HbA1c at 12 months, in persons with T1D with moderately impaired glycemic control. An FBA may, tentatively, have beneficial effects on food choices and nutrient quality. The results point toward several options in terms of nutrition programs to achieve health goals and quality of life and, thus, more ways to tailor the nutritional management of T1D to each individual’s needs and preferences.

## Data Availability

Data are available on reasonable request. Data consist of deidentified participant data. Contact person is Mette Axelsen, email: axelsen.nutrition@gmail.com
