# Supplementary material for: Effects of nutrition education using a food-based approach, carbohydrate counting or routine care in type 1 diabetes: 12 months prospective randomized trial
Source: BMJ Open Diabetes Res Care. 2021 Mar 31;9(1):e001971. doi: 10.1136/bmjdrc-2020-001971 (PMC8016079; doi:10.1136/bmjdrc-2020-001971)
Supplement: Supplementary data [file bmjdrc-2020-001971supp001.pdf]

Supplemental Table 1. Inclusion- and exclusion criteria

Inclusion criteria	Exclusion criteria
Adults (20-70 years)	Nephropathy (dU albumin>300 mg/d)
Type 1 Diabetes diagnose > 3 years	Retinopathy (grade>2)
BMI ≤ 35 kg/m²	Foot ulcers
Multiple dose insulin injection (MDI) therapy or pump insulin therapy	Gastroparesis
HbA1c 57-78 mmol/mol (7.4 - 9.3 % NGSP standard)	Diseases or conditions that affects food habits, metabolic control or compliance other than type 1 diabetes
Otherwise healthy	Current or planned pregnancy during the study period
Influence over their food and cooking	Obstacles to visit the hospital
Ability of oral and written communication in Swedish	Previous participation in carbohydrate counting education (≥ 4 hours during the last two years)
Written informed consent	Planning ≥ 14 days of fasting during the study period
	No computer access
