# Supplementary material for: Effects of nutrition education using a food-based approach, carbohydrate counting or routine care in type 1 diabetes: 12 months prospective randomized trial
Source: BMJ Open Diabetes Res Care. 2021 Mar 31;9(1):e001971. doi: 10.1136/bmjdrc-2020-001971 (PMC8016079; doi:10.1136/bmjdrc-2020-001971)
Supplement: Supplementary data [file bmjdrc-2020-001971supp002.pdf]

Supplemental Table 2. Food portfolio recommended in the Food Based Approach group.

Food group	Recommendation of daily intake	Examples
Vegetables and root vegetables	4 portions or more	All kinds of vegetables and root vegetables (not including potatoes)
Legumes	1 portion or more	Beans (soy, red, black, kidney, pinto), chickpeas, red, green and black lentils
Fruit and berries	2-3 portions	Apple, pear, citrus fruit, peach, nectarine, kiwi, plums and all kinds of berries. Other fruits *max 1 per day*
Nuts, almonds and seeds	1 portion (30 g)	All kinds of nuts and almonds. Sesame-, sunflower- and pumpkin seeds
Fish	2-3 portions/week or more	At least two portions of fatty fish and one portion of lean fish per week
Pasta, rice, grains, bread, cereals, muesli, porridge	Include in every meal	Choose high fiber whole grain products with low glycemic index
