# Supplementary figures and images for: Effects of nutrition education using a food-based approach, carbohydrate counting or routine care in type 1 diabetes: 12 months prospective randomized trial
Source: BMJ Open Diabetes Res Care. 2021 Mar 31;9(1):e001971. doi: 10.1136/bmjdrc-2020-001971 (PMC8016079; doi:10.1136/bmjdrc-2020-001971)

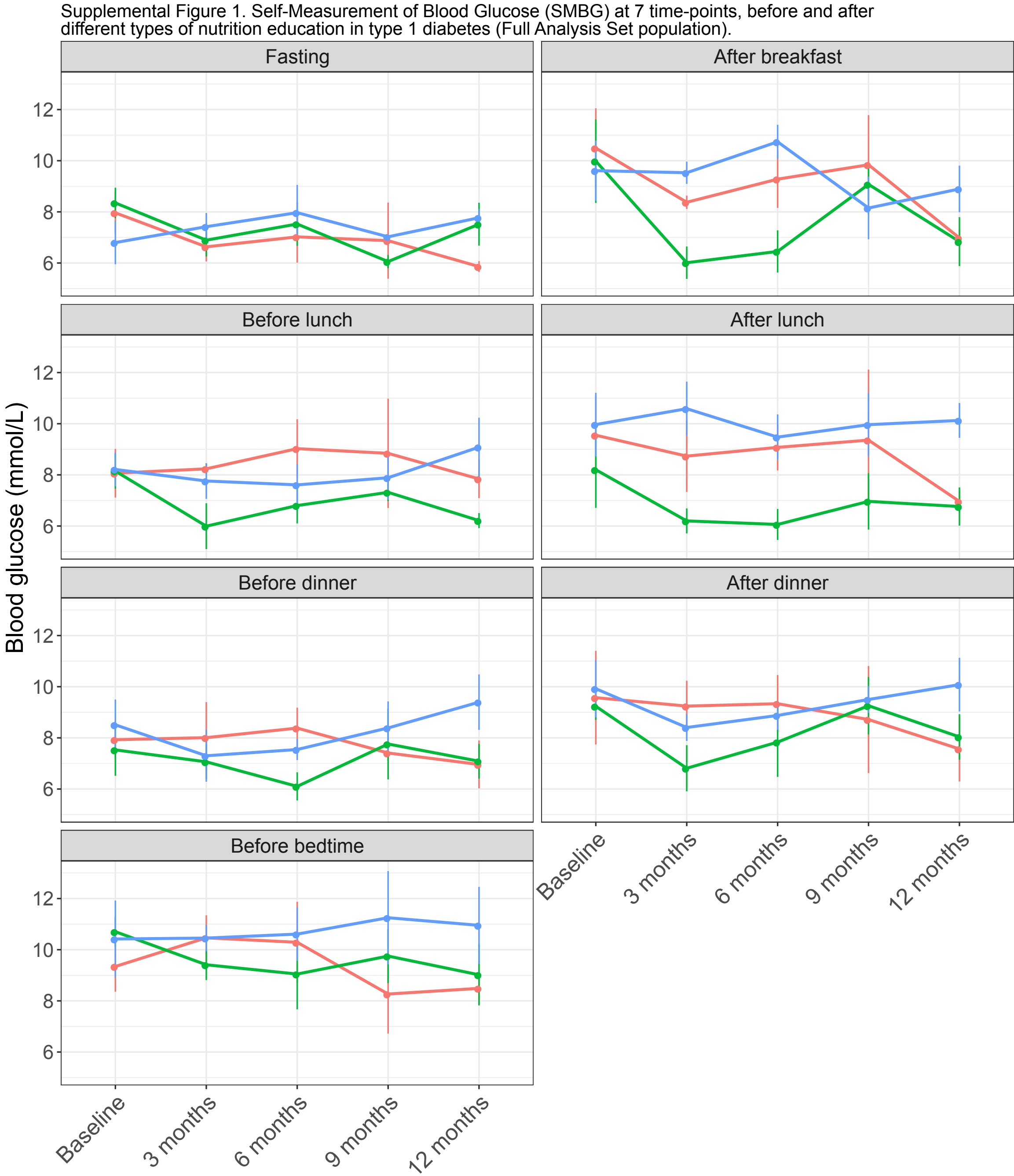

Data presented as means and 95 % CI. FBA=Food Based Advise, CC=Carbohydrate Counting, RC= Routine Care.

Supplement: Supplementary data [file bmjdrc-2020-001971supp003.pdf]
