# Supplementary material for: Effects of nutrition education using a food-based approach, carbohydrate counting or routine care in type 1 diabetes: 12 months prospective randomized trial
Source: BMJ Open Diabetes Res Care. 2021 Mar 31;9(1):e001971. doi: 10.1136/bmjdrc-2020-001971 (PMC8016079; doi:10.1136/bmjdrc-2020-001971)
Supplement: Supplementary data [file bmjdrc-2020-001971supp005.pdf]

Supplemental Table 3. Baseline data and differences in dietary intake between groups at baseline, 3 and 6 months in the Full Analysis Set

	Baseline			Month 3			Month 6		
	FBA[4]	CC[5]	RC[6]	FBA	CC	RC	FBA	CC	RC
Energy (kcal)	1636<br>(1321-2136)<br>n=42	1513[3]<br>(1328-1906)<br>n=41	1821[3]<br>(1472-2447)<br>n=41	0[2]<br>(-156-136)<br>n=42	0<br>(-327-147)<br>n=41	-181[2]<br>(-348-181)<br>n=41	0[2]<br>(-54-260)<br>n=42	67<br>(-333-329)<br>n=41	-144[2]<br>(-579-102)<br>n=41
Carbohydrates (g)	167[2]<br>(131-222)<br>n=42	177[3]<br>(126-207)<br>n=41	202[2,3]<br>(159-202)<br>n=41	-1<br>(-25-10)<br>n=42	0[3]<br>(-35-30)<br>n=41	-21[3]<br>(-42-2)<br>n=41	0<br>(-20-17)<br>n=42	0<br>(-51-39)<br>n=41	-12<br>(-63-11)<br>n=41
Protein (g)	71<br>(61-88)<br>n=42	67[3]<br>(56-82)<br>n=41	80[3]<br>(66-106)<br>n=41	0[2]<br>(-7-7)<br>n=42	-1<br>(-13-6)<br>n=41	-5[2]<br>(-18-0)<br>n=41	0[2]<br>(-5-13)<br>n=42	3[3]<br>(-14-12)<br>n=41	-9[2, 3]<br>(-27-0)<br>n=41
Fat (g)	65<br>(49-87)<br>n=42	55[3]<br>(48-73)<br>n=41	64[3]<br>(55-95)<br>n=41	0[2]<br>(-6-18)<br>n=42	-2<br>(-10-6)<br>n=41	-6[2]<br>(-16-0)<br>n=41	1[2]<br>(-4-15)<br>n=42	0[3]<br>(-11-15)<br>n=41	-6[2, 3]<br>(-23-1)<br>n=41
SFA (g)	25<br>(17-33)<br>n=42	21<br>(19-29)<br>n=41	24<br>(19-34)<br>n=41	0<br>(-6-4)<br>n=42	0<br>(-3-2)<br>n=41	-2<br>(-6-0)<br>n=41	0[2]<br>(-4-3)<br>n=42	1[3]<br>(-4-5)<br>n=41	-2[2, 3]<br>(-9-0)<br>n=41
MUFA (g)	24[1]<br>(18-32)<br>n=42	20[1, 3]<br>(18-27)<br>n=41	24[3]<br>(21-33)<br>n=41	0[1,2]<br>(-1-7)<br>n=42	-1[1]<br>(-4-3)<br>n=41	-3[2]<br>(-6-0)<br>n=41	0[2]<br>(-1-6)<br>n=42	0<br>(-6-5)<br>n=41	-3[2]<br>(-9-1)<br>n=41
PUFA (g)	11<br>(7-15)<br>n=42	10<br>(8-15)<br>n=41	12<br>(9-18)<br>n=41	2[1, 2]<br>(0-7)<br>n=42	0[1]<br>(-3-1)<br>n=41	0[2]<br>(-3-1)<br>n=41	1[1, 2]<br>(0-5)<br>n=42	0[1]<br>(-3-3)<br>n=41	0[2]<br>(-4-1)<br>n=41
n-3 (g)	0.2<br>(0.2-0.5)<br>n=42	0.2<br>(0.1-0.5)<br>n=41	0.2<br>(0.1-0.5)<br>n=41	0.0<br>(0.0-0.1)<br>n=42	0.0<br>(-0.1-0.1)<br>n=41	0.0<br>(-0.1-0.1)<br>n=41	0.0<br>(-0.1-0.1)<br>n=42	0.0<br>(-0.1-0.1)<br>n=41	0.0<br>(-0.1-0.1)<br>n=41
Sucrose (g)	26<br>(17-36)<br>n=42	27<br>(19-40)<br>n=41	30<br>(19-45)<br>n=41	0<br>(-3-3)<br>n=42	0<br>(-5-8)<br>n=41	0<br>(-12-2)<br>n=41	0[2]<br>(-5-7)<br>n=42	1<br>(-5-9)<br>n=41	-1[2]<br>(-14-5)<br>n=41
Fiber (g)	21[2]<br>(15-25)<br>n=42	19<br>(14-27)<br>n=41	23[2]<br>(18-23)<br>n=41	2[1, 2]<br>(0-7)<br>n=42	-1[1]<br>(-5-3)<br>n=41	-2[2]<br>(-5-0)<br>n=41	2[2]<br>(-1-7)<br>n=42	1<br>(-7-5)<br>n=41	-1[2]<br>(-5-2)<br>n=41
Wholegrain (g)	49<br>(26-73)<br>n=42	54<br>(28-74)<br>n=40	65<br>(39-99)<br>n=41	0[1]<br>(-1-22)<br>n=42	-4[1]<br>(-17-13)<br>n=40	0<br>(-16-10)<br>n=41	0<br>(-4-25)<br>n=42	-1<br>(-21-20)<br>n=40	0<br>(-18-6)<br>n=41
Legumes (portions/day)	0.0<br>(0.0-0,2)	0.0<br>(0.0-0.3)	0.0<br>(0.0-0.3)	0.0[1, 2]<br>(0.0-1.3)	0.0[1]<br>(0.0-0.0)	0.0[2]<br>(0.0-0.0)	0.3[1, 2]<br>(0.0-0.6)	0.0[1]<br>(0.0-0.0)	0.0[2]<br>(0.0-0.0)

	n=42	n=47	n=50	n=42	n=47	n=50	n=42	n=47	n=49
Nuts, seeds and almond (portions/day)	0.3<br>(0.0-0.6)<br>n=42	0.5[3]<br>(0.0-0.9)<br>n=47	0.1[3]<br>(0.0-0.5)<br>n=50	0.0 [1, 2]<br>(0.0-0.5)<br>n=42	0.0 [1]<br>(0.0-0.5)<br>n=47	0.0 [2]<br>(-0.3-0.0)<br>n=50	0.0 [1, 2]<br>(0.0-0.8)<br>n=42	0.0 [1]<br>(-0.1-0.3)<br>n=46	0.0 [2]<br>(-0.2-0.0)<br>n=50
Vegetables and root vegetables (portions/day)	2.1<br>(1.4-2.3)<br>n=42	1.8<br>(1.0-2.3)<br>n=47	2.0<br>(1.0-2.9)<br>n=49	0.0 [1]<br>(0.0-5.7)<br>n=42	0.0 [1]<br>(-0.9-1.8)<br>n=47	0.0<br>(0.0-2.2)<br>n=49	2.2 [1, 2]<br>(0.0-8.8)<br>n=42	0.0 [1]<br>(-3.5-1.8)<br>n=47	0.0[2]<br>(-3.5-0.9)<br>n=49
Fruit and berries (portions/day)	1.1<br>(0.9-1.5)<br>n=42	1.3<br>(0.8-1.8)<br>n=46	1.0<br>(0.5-2.0)<br>n=50	0.0<br>(0.0-0.3)<br>n=42	0.0<br>(0.0-0.3)<br>n=46	0.0<br>(0.00-3)<br>n=50	0.1 [1, 2]<br>(0.0-0.8)<br>n=42	0.0 [1]<br>(-0.5-0.5)<br>n=46	0.0 [2]<br>(-0.7-0.2)<br>n=50
Fish (portions/day)	0.4<br>(0.2-0.5)<br>n=42	0.3<br>(0.0-0.5)<br>n=47	0.3<br>(0.2-0.6)<br>n=50	0.0<br>(0.0-0.3)<br>n=42	0.0<br>(0.0-1.3)<br>n=47	0.0<br>(0.0-0.0)<br>n=50	0.1 [2]<br>(0.0-0.3)<br>n=42	0.0 [3]<br>(0.0-0.3)<br>n=47	0.0<br>(-0.3-0.0) [2, 3]<br>n=50
Wholegrain products (portions/day)	2.0<br>(1.3-3.0)<br>n=42	1.5<br>(1.0-2.8)<br>n=47	2.0<br>(1.0-3.1)<br>n=50	0.0<br>(-7.0-0.0)<br>n=42	0.0<br>(-3.5-0.0)<br>n=47	0.0<br>(-4.0-0.0)<br>n=50	-1.8<br>(-8.8-0.0)<br>n=42	0.0<br>(-3.5-0.0)<br>n=47	0.0<br>(-7.0-0.0)<br>n=50

Baseline data and differences in dietary intake between groups in FAS analysis expressed in medians and quartiles 1 and 3 at baseline and after 3 and 6 months. [1] FBA vs CC = p< 0.05, [2] FBA vs RC = p < 0.05, [3] CC vs RC p= < 0.05. [4] FBA=Food Based Advise group, [5] CC=Carbohydrate Counting group, [6] RC= Routine Care group.
