# Supplementary material for: Effects of nutrition education using a food-based approach, carbohydrate counting or routine care in type 1 diabetes: 12 months prospective randomized trial
Source: BMJ Open Diabetes Res Care. 2021 Mar 31;9(1):e001971. doi: 10.1136/bmjdrc-2020-001971 (PMC8016079; doi:10.1136/bmjdrc-2020-001971)
Supplement: Supplementary data [file bmjdrc-2020-001971supp006.pdf]

BMJ Publishing Group Limited (BMJ) disclaims all liability and responsibility arising from any reliance placed on this supplemental material which has been supplied by the author(s)

Supplemental Table 4. Baseline data and differences in dietary intake between groups expressed in medians and quartiles 1 and 3, PP population at baseline and after 3, 6 and 12 months.

	Baseline			Month 3			Month 6			Month 12		
	FBA[1]	CC[2]	RC[3]	FBA	CC	RC	FBA	CC	RC	FBA	CC	RC
Energy (kcal)	1627 (1303-2052) n=33	1497[6] (1350-1918) n=35	1821[6] (1416-2261) n=35	11[4] (-169-301) n=30	-16 (-362-168) n=34	-211[4] (-436-69) n=34	38[4] (-138-305) n=31	67 (-349-336) n=33	-224[4] (-672-119) n=33	70 (-243-225) n=31	-133 (-324-63) n=34	-110 (-657-124) n=34
Carbohydrates (g)	164 (134-217) n=33	173 (126-207) n=35	202 (149-254) n=35	-11[4] (-31-36) n=30	-6 (-34-35) n=34	-27[4] (-53-8) n=34	-4 (-29-19) n=31	-3 (-51-43) n=33	-15 (-82-15) n=35	1 (-30-20) n=31	-12 (-50-9) n=34	-23 (-79-18) n=34
Protein (g)	70 (60-85) n=33	64[6] (56-81) n=35	80[6] (65-95) n=35	3[4] (-8-11) n=30	-2 (-14-8) n=34	-9[4] (-18-2) n=34	2[4] (-9-13) n=31	5[5] (-14-12) n=33	-10[4, 5] (-32-2) n=33	1 (-8-13) n=31	-4 (-12-5) n=34	-6 (-29-6) n=34
Fat (g)	61 (48-84) n=33	55 (48-73) n=35	63 (55-90) n=35	4[4] (-8-22) n=30	-2 (-10-7) n=34	-7[4] (-17-3) n=34	3[4] (-4-16) n=31	3[5] (-13-14) n=33	-11[4, 5] (-27-4) n=33	4 (-4-14) n=31	-5 (-12-7) n=34	-4 (-26-10) n=34
SFA (g)	22 (17-30) n=33	22 (19-28) n=35	24 (19-34) n=35	-2 (-7-6) n=30	0[5] (-3-3) n=34	-2[5] (-8-0) n=34	1 (-5-3) n=31	1[5] (-4-5) n=33	-5[5] (-11-0) n=33	-1 (-6-3) n=31	-3 (-4-3) n=34	-2 (-13-2) n=34
MUFA (g)	23 (18-30) n=33	20[6] (18-27) n=35	24[6] (21-32) n=35	1[4] (-2-9) n=30	-2 (-4-4) n=34	-3[4] (-8-0) n=34	1[4] (-4-6) n=31	0 (-7-4) n=33	-5[4] (-11-1) n=33	1[4, 6] (-3-5) n=31	-2[6] (-5-2) n=34	-1[4] (-9-3) n=34
PUFA (g)	9 (7-16) n=33	10 (7-16) n=35	11 (9-16) n=35	4[4, 6] (0-9) n=30	-1[6] (-3-2) n=34	-1[4] (-3-2) n=34	3[4, 6] (1-6) n=31	0[6] (-3-2) n=33	-1[4] (-5-1) n=33	2[4, 6] (-1-6) n=31	-1[6] (-3-2) n=34	-1[4] (-5-2) n=34
n-3 (g)	0.2 (0.2-0.5) n=33	0.2 (0.1-0.5) n=35	0.2 (0.1-0.4) n=35	0.0 (-0.1-0.1) n=30	0.0 (-0.1-0.2) n=34	0.0 (-0.1-0.1) n=34	0.0 (-0.1-0.1) n=31	0.0 (-0.1-0.1) n=33	0.0 (-0.1-0.0) n=33	0.0 (-0.1-0.0) n=31	0.0 (-0.1-0.0) n=33	0.1 (-0.1-0.1) n=34
Sucrose (g)	26 (18-35)	27 (20-41)	30 (18-45)	2 (-4-7)	-1 (-5-6	-1 (-13-3)	-1 (-7-10)	1 (-7-5)	-3 (-14-5)	1 (-5-10)	0 (-7-3)	-2 (-16-3)

	n=33	n=35	n=35	n=30	n=34	n=34	n=31	n=33	n=33	n=31	n=34	n=34
Fiber (g)	21 (15-24) n=33	19 (14-28) n=35	21 (17-30) n=35	5[4,6] (-1-8) n=30	-2[6] (-5-4) n=34	-3[4] (-5-0) n=34	4[4,6] (-1-11) n=31	2[6] (-7-6) n=33	-1[4] (-5-3) n=33	1[4,6] (-3-9) n=31	-2[6] (-4-1) n=34	0[4] (-7-3) n=34
Wholegrain (g)	48 (25-71) n=33	54 (26-76) n=34	49 (35-96) n=35	8 (-16-25) n=30	-4 (-17-15) n=33	0 (-24-10) n=34	7[4] (-10-27) n=31	1 (-17-22) n=32	-2[4] (-21-6) n=33	5 (-25-21) n=31	-5 (-26-10) n=33	8 (-32-21) n=34
Legumes (portions/day)	0.0 (0.0-1.3) n=36	0.0 (0.0-0.3) n=41	0.0 (0.0-0.3) n=43	0.1[4, 6] (0.0-0.8) n=36	0.0[6] (0.0-0.0) n=41	0.0[4] (0.0-0.0) n=43	0.4[4, 6] (0.0-0.8) n=36	0.0[6] (0.0-0.0) n=41	0.0[4] (0.0-0.0) n=42	0.3[4, 6] (0.0-0.7) n=36	0.0[6] (0.0-0.3) n=41	0.0[4] (0.0-0.3) n=43
Nuts, seeds and almond (portions/day)	0.3 (0.0-0.8) n=36	0.5[6] (0.0-0.9) n=41	0.1[6] (0.0-0.5) n=43	0.0[4, 6] (0.0-0.5) n=36	0.0[6] (-0.4-0.0) n=41	0.0[4] (-0.3-0.0) n=43	0.3[4, 6] (0.0-0.8) n=36	0.0[6] (-0.2-0.3) n=40	0.0[4] (-0.3-0.0) n=36	0.3[4, 6] (0.0-0.8) n=36	0.0[6] (-0.5-0.2) n=40	0.0[4] (-0.3-0.3) n=43
Vegetables and root vegetables (portions/day)	2.1 (1.4-2.8) n=36	1.9 (1.0-2.5) n=41	1.9 (1.0-3.1) n=42	0.5[4] (0.0-1.4) n=23	0.0 (-0.4-0.6) n=25	0.0[4] (-0.5-0.5) n=27	0.8[4, 6] (0.0-1.3) n=27	0.0[6] (-0.9-0.4) n=30	-0.1[4] (-0.8-0.2) n=32	0.8[4] (-0.3-1.6) n=29	0.1 (-0.6-0.8) n=32	-0.3[4] (-1.0-0.3) n=30
Fruit and berries (portions/day)	1.1 (0.9-1.6) n=36	1.3 (0.8-1.9) n=40	1.0 (0.5-2.0) n=43	0.0 (-0.3-0.6) n=23	0.0 (-0.5-0.7) n=25	0.3 (-0.5-0.5) n=27	0.5[4,6] (0.0-1.0) n=27	0.0[6] (-0.5-0.4) n=30	0.0[4] (-0.9-0.4) n=32	0.3 (-0.1-0.9) n=30	0.0 (-0.3-0.7) n=32	0.3 (-0.1-0.7) n=30
Fish (portions/day)	0.3 (0.3-0.5) n=36	0.3 (0.0-0.5) n=41	0.3 (1.3-0.8) n=43	0.0 (0.0-0.3) n=23	0.0 (-0.3-0.3) n=25	0.0 (-0.3-0.3) n=27	0.3[4] (0.0-0.8) n=27	0.0 (-0.3-0.4) n=30	0.0[4] (-0.4-0.0) n=32	0.3 (-0.1-0.4) n=30	0.0 (-0.3-0.3) n=32	0.0 (-0.3-0.3) n=30
Wholegrain products (portions/day)	2.0 (1.2-3.0) n=36	1.5 (0.9-3.0) n=41	2.0 (1.0-3.0) n=43	-0.8 (-1.3-0.0) n=23	-0.3 (-0.7-0.2) n=25	-0.3 (-1.3-0.0) n=36	-1.0[6] (-1.5-0.3) n=27	-0.1[6] (-0.5-0.3) n=30	-0.3 (-1.4-0.0) n=32	-0.5 (-1.2-0.1) n=30	-0.4 (-1.2-0.2) n=32	-0.3 (-1.5-0.3) n=30

[1] FBA=Food Based Advise group, [2] CC=Carbohydrate Counting group, [3] RC= Routine Care group. [4] FBA vs CC, p< 0,05, [5] FBA vs RC, p< 0,05, [6] CC vs RC, p< 0,05.
