# Supplementary material for: Effects of nutrition education using a food-based approach, carbohydrate counting or routine care in type 1 diabetes: 12 months prospective randomized trial
Source: BMJ Open Diabetes Res Care. 2021 Mar 31;9(1):e001971. doi: 10.1136/bmjdrc-2020-001971 (PMC8016079; doi:10.1136/bmjdrc-2020-001971)
Supplement: Supplementary data [file bmjdrc-2020-001971supp007.pdf]

Supplemental table 5. Sensitivity analysis. Differences in HbA1c between the groups in the Full Analysis Set.

Time point	FBA[1] vs RC[2]	CC[3] vs RC	FBA vs CC
**3 months**			
mmol/mol	-0.8 (1.7) p=0.638	-3.4 (1.5) p=0.029	2.6 (1.8) p=0.168
NGSP %	0.1 (0.2)	0.3 (0.1)	0.2 (0.2)
**6 months**			
mmol/mol	0.0 (1.7) p=0.977	-2.1 (1.5) p=0.158	2.1 (1.8) p=0.248
NGSP %	0.0 (0.2)	0.2 (0.1)	0.2 (0.2)
**9 months**			
mmol/mol	-0.3 (1.2) p=0.793	-1.0 (1.2) p=0.394	0.5 (1.2) p=0.663
NGSP %	0.0 (0.1)	-0.1 (0.1)	0.1 (0.1)
**12 months**			
mmol/mol	0.6 (1.9) p=0.765	-1.2 (1.5) p=0.438	1.8 (1.8) p=0.341
NGSP %	0.1 (0.2)	0.1 (0.1)	0.2 (0.2)

Data shown as means and (SD). [1]FBA=Food based approach, [2]RC=Routine Care, [3]CC= Carbohydrate counting.
